# Mesenchymal Stem Cells and Regenerative Medicine

**DOI:** 10.1155/2018/9810972

**Published:** 2018-10-29

**Authors:** Huseyin Sumer, Jun Liu, Sangho Roh

**Affiliations:** ^1^Department of Chemistry and Biotechnology, Faculty of Science, Engineering and Technology, Swinburne University of Technology, Burwood Road, Hawthorn VIC 3122, Australia; ^2^Stem Cells and Genome Editing, Agriculture Victoria Research Division, Department of Economic Development, Jobs, Transport and Resources (DEDJTR), AgriBio Centre, 5 Ring Road, Bundoora VIC 3083, Australia; ^3^Lab of Cellular Reprogramming and Embryo Biotechnology, School of Dentistry, Seoul National University, Gwanak-gu, Seoul 08826, Republic of Korea

Mesenchymal stem cells (MSCs) are multipotent stromal cells that are able to differentiate into a number of tissues such as bone, fat, cartilage, and muscle. They can be isolated from a number of sources, and because of their differentiation and immunomodulatory properties, they have relevant and significant clinical therapeutic applications. MSCs are currently being used to treat a number of clinical conditions, as well as being tested in preclinical and clinical trials around the world due to their tissue regenerative properties.

In this special issue, we have a number of primary research articles examining the potential clinical application or preclinical studies of MSCs or their derivatives. The topics that are covered include the isolation from different sources, derivation, differentiation, quality control, and transplantation studies involving MSCs. We also have a number of review articles relating to the abovementioned aspects of MSCs. This special issue summarizes the most recent developments in the field in the following areas:

- Advances in existing isolation, derivation, culture, or differentiation methods, in particular the development of scalable clinically relevant systems
- Maintenance, differentiation, transdifferentiation, and homing of behaviour of MSCs
- Biomaterials for culture, expansion, differentiation, and transplantation of MSCs
- Transplantation of MSCs into in vivo model systems of disease
- Development of good manufacturing practice protocols for clinical grade MSCs
- Transplantation studies of MSCs and the immune response after MSC transplantation

Forty papers were submitted for this special issue. Our distinguished reviewers from respective research fields narrowed the field to twenty-six which were finally accepted. The following is a short summary of the findings of each of these papers.

B. C. Bellagamba et al. revealed that specific culture conditions including IGF-1 can endow cultured MSCs with the expression of CD271 and CD34 and may enhance the multipotency of these cells when they are used for therapeutic purposes.

B. Follin et al. used a rat model of myocardial infarction to test the administration of adipose-derived stromal cells (ASCs) in a translational *in situ* forming alginate hydrogel and report a combination therapy of cell therapy and biomaterials.

M. S. Hu et al. provided a comprehensive review on the experimental applications of various stromal cells to promote wound healing and discussed the novel methods used to increase MSC delivery and efficacy.

M. Kuzma-Kozakiewicz et al. performed intraspinal transplantation of the adipose tissue-derived regenerative cells in amyotrophic lateral sclerosis in accordance with the current experts' recommendations to choose optimal monitoring tools and listed some suggestions for further clinical trials.

J. M. Lasso et al. proposed that adipose-derived regenerative stem cell (ADRC-) enriched autologous adipose tissue grafts can be used as an effective filler for the paralyzed vocal fold to use it for functional reconstruction of the glottal gap.

Y. Liu et al. demonstrated that mesenchymal stem cells enhance liver regeneration (LR) via improving lipid accumulation and Hippo signaling. They showed the potential of MSCs to improve LR through cell growth and cell proliferation.

M. Zhang et al. investigated the effect of donor age on the properties of human eyelid adipose-derived stem cells (HEASCs). HEASCs from a number of age groups were evaluated and quantified for their proliferative capacity, colony-forming ability, surface markers, differentiation ability, secreted proteins, and wound healing function.

A. Mizukam and K. Swiech reviewed the current MSC manufacturing platforms with special attention regarding the use of bioreactors for the production of GMP compliant clinically relevant cell numbers. They also addressed first commercial MSC-based products.

M.-Y. Moon et al. investigated the effect of zinc, an essential element required for cell division, migration, and proliferation, on the proliferation and differentiation of multipotent adipose-derived mesenchymal stem cells (AD-MSCs).

S. Nahar et al. compared the University of Wisconsin (UW) solution with Hank's balanced salt solution for the preservation of mouse adipose tissue-derived mesenchymal stem cells. They found that the number of viable cells harvested per gram of adipose tissue mass was higher in UW solution than HBSS-preserved tissue.

N. Petinati et al. suggested that MSCs could participate in the restoration of niches for donor hematopoietic cells or have an immunomodulatory effect, preventing repeated rejection of the graft, and perhaps intraosseous implantation of MSCs contributes to the success of the second transplantation of hematopoietic stem cells and patient survival.

S. Nepali et al. compared human adipose-derived mesenchymal stem cells from orbit (orbital-ASCs) and abdomen (abdominal-ASCs) and concluded that tissue-harvesting site is a strong determinant for characterisation of adipose-derived mesenchymal stem cells as the expression level of some surface markers is different between two sites, orbit and abdomen.

S. Niada et al. implemented differential proteomics to investigate secretome of adipose-derived mesenchymal stem/stromal cells and focused on the potential applications in regenerative medicine. They showed the neuroprotective action of conditioned media from MSCs and an unexpected proosteogenic aptitude of conditioned media from dermal fibroblasts.

E. Ø. Nielsen et al. harvested adipose tissue from ovine and verified isolated cells for minimal criteria for adipose stromal cells which suggests a feasible method for isolation of ovine ADSCs. Moreover, osteogenic induction medium (OIM) containing fibroblast growth factor basic (FGFb), bone morphogenetic protein 2 (BMP2), or NEL-like molecule 1 (NELL1) showed significantly higher positive Alizarin red staining cells to the basic medium.

N. Petinati et al. present the results of from patients after hematopoietic stem cell transplantation with simultaneous implantation of MSCs from their respective donors into cancellous bone. The results suggest that MSCs could participate in the restoration of stem cell niches for donor hematopoietic cells or have an immunomodulatory effect, preventing repeated rejection of the graft.

I. Roato et al. focused on adipose tissue-derived stromal vascular fraction as an abundant source of mesenchymal stem cells as an alternative source for bone regeneration. They report the use of SmartBone as an effective bone biological substitute, combined with adipose-derived stromal vascular fraction as potential instrument for the treatment of bone defects.

M. Seo et al. reported that conditioned media of mesenchymal stem cells (MSCs) isolated from deer antlers enhanced tissue regeneration through paracrine action via a combination of secreted growth factors and cytokines. The work also showed that the use of deer antler MSC-conditioned media (DaMSC-CM) as a unique natural model for rapid and complete tissue regeneration has possible application for therapeutic development.

K. Shah et al. review the current treatment options for osteoarthritis (OA) and the exciting new translational medical research currently underway utilising mesenchymal stem cells for OA therapy.

K. Shah et al. report the outcome of an extensive study of the therapeutic use of allogeneic stem cell therapy in osteoarthritic dogs. They present the outcome in safety and efficacy of allogeneic adipose-derived mesenchymal stem cells derived from healthy donor dogs in treating osteoarthritic dogs with lameness and suffering from pain.

T. Sultana et al. review the therapeutic application and scope of umbilical cord blood-derived mesenchymal stem cells (UCB-MSCs) that can be isolated from different species with a particular focus on canine UCB-MSCs.

D. N. Silva et al. investigated function of including insulin-like growth factor-1 (IGF-1) in the IGF-1-overexpressing MSCs (MSC_IGF-1) for the treatment of diseases that affect the cardiovascular system, including Chagas disease. Their results indicate the therapeutic potential of MSC_IGF-1, with combined immunomodulatory and proregenerative actions to the cardiac and skeletal muscles.

A. D. Veron et al. demonstrated, for the first time, that syngeneic transplantations of olfactory ectomesenchymal stem cells (OE-MSCs) in rats can restore cognitive abilities impaired after brain injuries and provide support for the development of clinical studies based on grafts of OE-MSCs in amnesic patients following brain injuries.

M. Viganò et al. provided a comprehensive article on the strategies and the results of validation studies applied to GMP quality control methods for the clinical use of mesenchymal stromal cells (MSC). They provide a useful tool to face the hurdles in the application of the rules regulating the quality control of cell therapy medicinal products such as MSC.

K. Ye et al. investigate the use of endometrial regenerative cells (ERCs) in aorta allografts in mouse models and found that overexpression of B7-H1, also known as programmed cell death-1 ligand (PD-L1), stimulated by IFN-*γ* is required for ERCs to prevent the transplant vasculopathy.

W. Zhun et al. investigated the efficiency of the allograft bone marrow stem cells (BMSCs) with Dkk-1 interference in preventing the progress of the rat model for hip disorder glucocorticoid-induced osteonecrosis of the femoral head. They showed that lentivirus-meditated Dkk-1 RNAi resulted in the promotion of osteogenesis and inhibition of adipogenesis. The progression of the disease was prevented; however, the effects were not significantly superior to treatment with nongenetically modified or normal BMSCs.

K. Ye et al. demonstrated that B7-H1 expression on endometrial regenerative cells (ERCs) was upregulated by IFN-*γ* in a dose-dependent manner, and it was required for ERCs to inhibit the proliferation of peripheral blood mononuclear cells (PBMCs) in vitro. This study provides a theoretical basis for the future clinical use of human ERCs.

M. Zayed et al. investigated the *in vivo* potential of synovial fluid-derived mesenchymal stem cells and bone marrow-derived MSCs in articular cartilage repair. They performed xenogenic implantation of equine MSCs in a rat osteochondral defect model and evaluated articular cartilage healing.

B. Zhang et al. observed significantly higher improvement in cartilage neogenesis and recovery using canine umbilical cord mesenchymal stem cells (UC-MSCs) on treatment of knee osteoarthritis in dogs. The joint fluid and the inflammatory response decreased. Moreover, improved recovery in the neogenetic cartilage, damaged skin fascia, and muscle tissue around the joints was more significant in the treated group.

